# Concomitant bilateral popliteal arterial aneurysm and abdominal aortic aneurysm in a case with severe occlusive disease of lower extremity

**DOI:** 10.1186/1749-8090-8-S1-P90

**Published:** 2013-09-11

**Authors:** U Yetkin, I Peker, H Iner, I Yurekli, A Gurbuz

**Affiliations:** 1 Department of Cardiovascular Surgery, Izmir Katip Celebi University Ataturk Training and Research Hospital, Izmir, Turkey

## Background

Aneurysmal dilation of popliteal artery's diagnosis is quite difficult due to its insidious and asymptomatic course although it consists of 2/3 of all peripheral arterial aneurysms.

## Methods

Our case was a 53-year-old male. He was presented to our surgical council after detailed investigations carried out at another institution. His past medical history was significant for left infragenual femoropopliteal bypass using PTFE graft 2 years ago. He was complaining from claudication of his left lower extremity at a walking distance of 100 m.

## Results

Color Doppler ultrasound revealed bilateral popliteal arterial aneurysm of 2-2.5 cm. Computed tomographic angiography showed a fusiform aneurysmal dilation of the abdominal aorta with a largest diameter of 38 mm. Left superficial femoral artery was totally occluded and the graft was patent. There was a high-grade stenosis at the site of proximal anastomosis. Both popliteal arteries showed aneurysmal dilation of 35 and 25 mm, right and left sides, respectively. Right peroneal artery was patent but the remaining two branches were perfused via collaterals. All of the 3 infragenual branches were patent on left with occlusion of the anterior tibial artery distally. Pedal arteries were patent. Coronary angiogram discovered some non-significant stenotic lesions at left anterior descending and right coronary arteries. With these findings, we planned percutaneous balloon dilation or, if necessary, stenting of the proximal anastomosis site and endovascular stent grafting of both popliteal arteries. He preferred to remain in outpatient follow-up.

## Conclusions

Popliteal arterial aneurysms may be seen bilaterally and coexistent abdominal aortic aneurysm may be detected in 50% of cases. The priority in the treatment of the aneurysm includes prevention of limb ischemia or limb loss. In addition to the open surgical approach, endovascular stent grafting gains popularity and becomes a widely performed application.

